# Supplementary material for: More to diverse: Generating diversified responses in a task oriented multimodal dialog system
Source: PLoS One. 2020 Nov 5;15(11):e0241271. doi: 10.1371/journal.pone.0241271 (PMC7644051; doi:10.1371/journal.pone.0241271)
Supplement: Attachment — Submitted filename: Response-file-Feb2020.pdf [file pone.0241271.s002.pdf]

Response to Comments of the Reviewers

February 29, 2020

1 Response to Editor's Comments

This paper has been revised based on the feedbacks of the Editor, Associate Editor and/or Reviewers. In the revised version, we have tried to address all the concerns in the best possible ways. The comments and/or feedbacks were all very helpful, and we are appreciative of such constructive suggestions on our previous submission. We respond to the comments of the reviewers point-wise. We hope the revisions would improve the quality of our paper.

The major changes incorporated in the current paper are enumerated as follows:

- In the revised version of the manuscript, we have very carefully reviewed the article and resolved all the grammatical mistakes and typos.
- We have included the rationale behind our current work by highlighting the very specific problem that we solve in natural language generation.
- We have incorporated the detailed comparisons of our present work to the existing state-of-the-art methods.
- In the revised article, we have included more descriptions of the evaluation metrics.
- As suggested, we have now got our paper proofread for grammar and English by Dr. Abhinav Tripathay, linguist in our research group.

Comment 1: Please explain the rationale for the development of your system in light of recent research in this area, clearly indicating which problem with existing systems you are addressing.

Response: The explanation for the development of the current system is provided in Section 1.1 and Section 1.2 of the manuscript highlighting the problem definition and motivation of the current work. In this work, we address the task of generating diverse responses in a multimodal dialogue setup capturing information from both text and images. Generating diverse responses is an important research direction in dialogue generation. Recently researchers have developed robust neural frameworks for generating interesting and interactive responses in unimodal (textual) dialogue systems. With the advancement in

artificial intelligence the research in dialogue systems have taken a step ahead by bringing different modalities such as images, audio and videos together for building robust systems. Hence, in our present work, we focus on generating diverse responses in a multimodal dialogue system. The second important characteristics of our model is to incorporate the politeness while generating the response.

Comment 2: Please clearly report at the beginning of your methods or results section which were the key performance measures used to establish the validity and utility of your system. Please also report clearly which statistical analysis was used to establish robustness of performance measures.

Response: As suggested we have reported the evaluation metrics used for measuring the performance of the model at the beginning of the results section. In Section 6.1 of the manuscript, we employ both automatic and human metrics to measure the performance of the proposed framework. In case of automatic metrics we report the efficacy of our proposed method in terms of BLEU, Perplexity, distinct-1, distinct-2 and politeness accuracy. For proper and complete evaluation of the system we also perform human evaluation of the responses generated by our proposed framework. We report fluency, diversity and politeness metrics for measuring the grammatical correctness, diverseness and politeness of the generated responses, respectively. For statistical significance we report Welch's t-test.

Comment 3: Whilst we note that the performance of your system has been compared to other methods, the manuscript is lacking a thorough discussion of the methods chosen for comparison and justification as to why they were chosen. Please modify your manuscript as necessary. Please note that PLOS ONE requires that experiments, statistics, and other analyses must be performed to a high technical standard and described in sufficient detail to allow for reproducibility of the study. To demonstrate the performance of the system, we would expect comparisons to be drawn between existing state-of-the-art methods.

Response: Detailed discussions on the comparisons between the existing systems and our current work are provided in Section 5.2 of the manuscript. We have provided the implementation details of our system in Section 4.1 of the manuscript for the reproducibility of our work. Also the codes will be made available in due time.

Comment 4: Additionally, we asked that you clearly state in the methods section where you accessed the data confirm that you have followed the terms and conditions of the use of the data in the study.

Response: For the experimentation, we have used the recently released Multimodal dialog dataset [Saha et. al., 2018]. The data has been created for response generation in a multiodal setup. We have followed the terms and usage of the data in our work. Also, the terms and conditions for the usage of the dataset are provided in Section 3.

Comment 5: Furthermore, if your manuscript contains content such as photos, images, figures, tables, audio files, videos, etc., that you or your co-authors do

not own, we will require you to provide us with proof that the owner of that content (a) has given you written permission to use it, and (b) has approved of the CC BY license being applied to their content. If you are unable to provide this, we ask that you remove such images from the manuscript and replace them with CC-BY images.

Response: In the manuscript all the images, figures and tables belong to the authors only and are not copied or taken from any other source.
