# Supplementary material for: More to diverse: Generating diversified responses in a task oriented multimodal dialog system
Source: PLoS One. 2020 Nov 5;15(11):e0241271. doi: 10.1371/journal.pone.0241271 (PMC7644051; doi:10.1371/journal.pone.0241271)
Supplement: Attachment — Submitted filename: Answer_file_PLoS.pdf [file pone.0241271.s003.pdf]

Response to Comments of the Editor(s) and Reviewers

July 21, 2020

1 Response to Editor's Comments

We must thank the Editor, Associate Editor and Reviewers for their insightful suggestions and feedbacks. This paper has now been revised based on the feedbacks of the Editor, Associate Editor and/or Reviewers. In the revised version, we have tried to address all the concerns in the best possible ways. The comments and/or feedbacks were all very helpful, and we are appreciative of such constructive suggestions on our previous submission. We respond to the comments of the reviewers point-wise. We hope the revisions would improve the quality of our paper.

The major changes incorporated in the current paper are enumerated as follows:

- In the revised version of the manuscript, we have very carefully reviewed the article and resolved all the grammatical mistakes and typos.
- We have included the rationale behind our current work by highlighting the very specific problem that we solve in natural language generation and the motivation of every component used in the methodology.
- We have added the suggested references to provide a comprehensive survey of the existing literature.
- We have incorporated the detailed comparisons of our present work to the existing state-of-the-art methods.
- In the revised article, we have included more implementation details of the proposed method and the classifier used for evaluation.
- As suggested, we have now got our paper proofread for grammar and English very thoroughly. We have also got it checked by Dr. Abhinav Tripathay, linguist in our research group.

2 Responses to the comments or queries of Reviewer 1

- **Query 1:** This research paper is based on the previously proposed multimodal system. Due to unimodal systems have got some problems in the dull and boring responses as authors indicated in the section 1.1 and 2.1, authors employed BLOCK fusion technique and used stochastic beam search with Gumble k-tricks to generate diversified responses (described in the section 2.2). Authors have described the methods for proposed multimodal system. However, authors have also got a mention in the Editor's Comment 3 that PLOS ONE requires experiments, statistics, and other analyses must be performed. I could not find any detail and illustration for the discussion of the section 5 Experiments. There is section 5.1 Implementation Details only. It is difficult to identify if this research is good or not.

  Response: We appreciate the comments of the reviewers. The implementation details provided in Section 5.1 are in accordance to the proposed framework described in the Methodology Section 3.2, 3.3 and 3.4 of the manuscript. We have also provided motivation and explanation of each component for better understanding in Section 3.3.1 and 3.3.2 of the revised article. Furthermore, the details of both automatic evaluation and human evaluation metrics have been provided for better understanding in Section 6.1.1 and Section 6.1.2, respectively.

  The proposed framework has been compared to the existing approaches in a detailed manner in Section 6.3 with respect to the results obtained in Table 2 (rows 1-5 and 13-14) of the revised manuscript. In addition, we have also compared the proposed framework with the different baselines (as described in Section 3.5) in Section 6.2 and Section 6.3 with comprehensive analysis in terms of both automatic and human evaluation. For better understanding, the statistics of the MMD dataset used for experimentation has been provided in Section 4, Table 1 of the manuscript. The changes incorporated in the data has also been included in the second paragraph of Section 4 with examples showcasing the changes in Figure 4 of the revised manuscript. We have incorporated more details of the politeness classifier in Section 5.1 of the revised version. The attention analysis with respect to the knowledge base (KB) inputs with more explanation has been presented in Figure 7, and in the first paragraph of Section 6.5 of the revised manuscript. In addition, for better analysis of the Parallel Co-attention, we have included the attention visualization in Figure 8 and provided a detailed description in the second paragraph of Section 6.7. Finally, we provide analysis of the errors encountered by the proposed framework, and show examples of each in Section 6.6 of the revised version.

3 Responses to the comments/queries of Reviewer 2

- **Query 1:** The definition of the problem is confusing on page 5. U_p,j, and I_p,j', what are the meanings of p, j, and j'? Why does history H_p just contain from (U_1, I_1) to (U_{p-1}, I_{p-1})? What are the roles of the user U_p and image I_p? More important, as shown in Fig. 1, in the dialogue, S1 sometimes answer with both images and text. But in the paper, the authors just generate the text answer. The author should clarify the definition of the multi-modal dialog task.

  Response: As advised by the reviewer we provide the clarification of the problem statement. In the problem definition, p is the pth turn of a given dialogue, while j is the number of words in a given textual utterance and j' is the number of images in a given utterance. Note that in every turn, the number of images $j' \leq 5$. Hence, for text, vectors of zeros are considered in place of image representation. The dialogue history H_p contains all the previous utterance information having both text and images from (U_1, I_1) to (U_{p-1}, I_{p-1}) to provide the essential context for the generation of the next textual response. The user utterances play a significant role in building the dialogue context for generating coherent and appropriate responses, in accordance to the user demands. In our current work, we focus on generating the textual responses only in a similar manner as Chauhan et. al. [8], Agarwal et. al. [12,13]. Here, the multi-modal dialog task corresponds to a setup that considers both the modalities, i.e. text and images by effectively combining these information for the generation of the textual response as opposed to uni-modal systems that typically uses only textual information for generating the next response. Please note that the primary objective of this work is to generate diversified and polite responses for increasing the human-machine interaction. Therefore, we consider only textual utterance of a dialog for the generation, and we do not focus on image retrieval or image generation in our current work.

  This information has been included in Section 1.1 (Problem Definition, last paragraph) of the revised manuscript.

- **Query 2:** The authors have already introduced and discussed many works related to NLG. To my knowledge, the multimodal dialog task is much closer to the image dialog task. The references related to the dialog task are insufficient. [1] Wu et al. Are you talking to me? Reasoned visual dialog generation through adversarial learning. In CVPR, 2018. [2] Guo et al. Dual visual attention network for visual dialog. In IJCAI, 2019. [3] Guo et al. Textual-Visual Reference-aware Attention Network for Visual Dialog. IEEE Transactions on Image Processing, 2020.

  Response: As suggested by the reviewers, we have incorporated these references in the Related Work (Section 2.2) of the revised manuscript. The added references are numbered as [36], [37], [38] in the revised manuscript.

- **Query 3:** The novelty is limited. For example, parallel Co-Attention (PCA), including the BLOCK fusion for multi-modal interaction, has been widely applied as a common attention framework. It is unclear about the motivation. Are there any more insights about your proposed model?

  Response: As suggested by the reviewer, we have included the motivation for Parallel Co-Attention (PCA) and BLOCK fusion in the first paragraph of the Methodology Section, i.e. Section 3.3.1 and in the first paragraph of Section 3.3.2, respectively. We would like to bring into the notice that parallel co-attention technique has not been employed for the related work, i.e. for the dialogue system. As opposed to visual question answering having single image, in multi-modal dialogue system we have multiple images over a context of turns. In our case, we apply parallel co-attention between the textual utterance and the multiple images present in the utterance to attain significant attributes, and accordingly generating the textual response. Since, parallel co-attention draws the essential features between the text and visual counterparts, hence in our work we employ PCA to obtain the important information from the text and the different images present in the input utterance. This facilitates the network in generating the attribute centered responses as the attention focuses on the different attributes, such as color, pattern, shape, etc. of the products. The attention network, in accordance to text, captures the appropriate image information from multiple images. For example, the attended image is in consonance to the text such as 3rd image, 2nd image, etc. By achieving the relevant image information the generated response becomes more coherent to the dialogue history. There is no previous literature that shows the use of parallel co-attention for building the dialogue systems.

  The motivation of BLOCK fusion technique has been incorporated in the first paragraph of Section 3.3.2 in the revised manuscript. Previous works on multi-modal dialogue systems have merely concatenated the textual and visual representation, thereby having only linear interaction between the modalities. This is often insufficient in capturing the complex interactions of different modalities. For better non-linear interaction between the two modalities, we apply BLOCK fusion technique. With recent success of BLOCK fusion technique in visual question answering, we incorporated it for multi-modal dialogue system where the interaction between the modalities is highly intricate as the previous dialogue history along with multiple images needs to be considered for generating the appropriate responses that are contextually coherent with both the textual and visual modalities.

- **Query 4:** Fonts in Fig.6 are too small.

  Response: As correctly suggested by the reviewers we have increased the font size of the Figure 6 for better readability and understanding.

- **Query 5**: The implementation details of the politeness classifier in Fig.5

should be given in the paper.

Response: As rightly advised by the reviewers we have incorporated the implementation details of the politeness classifier in the second paragraph of Section 5.1 of the revised manuscript. To measure the politeness quotient in the generated response we design a politeness classifier as presented in Figure 5. The input is first converted into embeddings using the embedding layer. We use 300-dimensional Glove embedding for representing the utterances. The embedded utterance is fed to the convolutional layer with filter size 3. After convolution, we apply max pooling to get the hidden representation. For enhanced representation, we apply a unidirectional LSTM network on the hidden representation followed by self-attention and obtain the final representation of the utterance. The number of neurons on the LSTM layer is fixed to be 200. Finally, we apply the softmax layer for attaining the politeness accuracy of the given utterance. To avoid over-fitting of the neural network a useful regularisation technique known as dropout has been used in our model. At the time of forward propagation, the neurons are randomly tuned-off so that the convergence of weights is restricted to identical positions. For optimisation and regularisation, we use Adam optimiser along with 15% dropout in our model. Categorical cross-entropy is employed to update the model parameters.

- **Query 6**: Compared with [8] and [38], the performance superiority of the proposed method is inapparent. The authors should give more discussion.

  Response: We appreciate the reviewer for this comment. Our proposed framework shows improvement from the existing systems, such as Chauhan et. al. [8] and Agarwal et. al. [13]. We include the complete analysis in comparison to the existing approaches in Section 6.3 of the revised manuscript. In Chauhan et. al. [8], authors employed position and attribute aware attention for capturing the significant information from both textual and visual part of the utterance, while Agarwal et. al. [13] utilized knowledge base for the generation of textual responses. By applying parallel co-attention and BLOCK fusion technique, our best performing baseline framework achieves an improvement of around 5% in BLEU score from both the existing approaches. This is mainly due to the fact that the parallel co-attention network simultaneously captures the essential information from the entire utterance possessing textual and visual knowledge. We also attain enhanced representation of the utterance by having improved interaction between the modalities with the help of BLOCK fusion technique. One of the purposes of our current research is to accomplish diverse response generation. Evaluation shows that by applying stochastic beam search, we observe an improvement of 2% in BLEU score (with k=5) as opposed to Chauhan et. al. [8] and Agarwal et. al. [13]. The increase in BLEU score signifies that the responses are diverse and different from the ground-truth responses, thereby, helping in

achieving the desired task. Also, the decrease in perplexity score testifies that the generated responses are grammatically correct and better in comparison to all the baselines and the existing frameworks.
