# Supplementary material for: More to diverse: Generating diversified responses in a task oriented multimodal dialog system
Source: PLoS One. 2020 Nov 5;15(11):e0241271. doi: 10.1371/journal.pone.0241271 (PMC7644051; doi:10.1371/journal.pone.0241271)
Supplement: Attachment — Submitted filename: Answer_file2_PLoS.pdf [file pone.0241271.s004.pdf]

Response to Comments of the Editor(s) and Reviewers

September 14, 2020

1 Response to Editor's Comments

We must thank the Editor, Associate Editor and Reviewers for their insightful suggestions and feedbacks. This paper has now been revised based on the feedbacks of the Editor, Associate Editor and/or Reviewers. In the revised version, we have tried to address all the concerns in the best possible ways. The comments and/or feedbacks were all very helpful, and we are appreciative of such constructive suggestions on our previous submission. We respond to the comments of the reviewer(s) point-wise. We hope the revisions would improve the quality of our paper.

The major changes incorporated in the current paper are enumerated as follows:

- In the revised version of the manuscript, we have added the analysis in the form of Tables and Figures with regards to the network parameters such as dropout, learning rate and LSTM layers.

2 Responses to the comments or queries of Reviewer 1

- **Query 1:** Authors have applied the parallel co-attention technique between the textual utterance and the multiple images present to generate significant attributes and the textual response. Although authors have done the research in the multimodal dialogue system with the parallel co-attention technique, it is still necessary to give some experimental details for this article. In the Section 5.1, authors provided a brief explanation for the implementation details. There are still some key points that authors did not present for the implementation details of experiments about the determination of network parameters as follow (1). The AMSGrad was be used as the model training optimizer, and dropout was be used to avoid over-fitting that refer to Hinton's publication in 2014. Why the probability of 0.4 was be determined for the model training? Please give

experimental details with the statistical analysis in the use of Table and Figure. (2). In the same questions to determination of the fixed learning rate of 0.0004 and LSTM layers of 200, Please give experimental details with the statistical analysis in the use of Table and Figure.

Response: We appreciate the comments of the reviewers. In Section 6.3 (Analysis of Network Parameters) of the revised manuscript we have added the complete analysis with respect to the dropout, learning rate and LSTM layers for model fine-tuning. We used Perplexity (PPL) as the primary metric to fine-tune the framework for determining the network parameters in case of dropout and learning rate. After fixing these parameters we do the complete evaluation in terms of both automatic and human metrics for all the baselines and proposed framework. We used politeness accuracy (PA) as the primary metric for determining the number of LSTM layers in the politeness classifier. The dropout probability was determined to be 0.4 by considering the range of 0.1 - 0.8. The statistical analysis of the results for the different values of dropout probability for the proposed framework is provided in Table 3 of the revised manuscript. In Figure 4(a) we depict the performance of the proposed framework in case of dropout. It is evident that the proposed framework performs best when the dropout probability is 0.4. Therefore, for all the experiments (all baselines) we fix the dropout probability as stated.

For learning rate we determined the value to be 0.0004, in a similar way as [12, 13, 44]. Also, we cross-verified by taking a range of 0.001 to 0.0001 to determine the learning rate in case of our proposed framework. As shown in Table 3 and Figure 6(b), the proposed framework performs best when the learning rate is fixed at 0.0004. This is in line with the existing literature.

In the last paragraph of Section 6.3, we provide the analysis for the LSTM layers in the classifier. For the politeness classifier, we had fixed the LSTM layers at 200 by evaluating the performance of the classifier for the responses generated by our proposed framework. We checked the performance of the classifier by using different layers of LSTM as shown in Table 4 of the revised manuscript. From the Figure 6(c), it is evident that by increasing the number of LSTM layers the performance of the classifier improved with the maximum accuracy being 0.84 in case of 200 layers. On increasing the number of layers the performance declined and eventually became constant with accuracy lesser than the best performing classifier at 200 layers. This analysis helped in deciding the number of LSTM layers for the classifier.
